# Hospital Finance and Economy.—III

**Published:** 1888-11-10

**Authors:** 


### HOSPITAL FINANCE AND ECONOMY.—III.

BY A MEDICAL SUPERINTENDENT.

(*Continued from page* 20.)

### THE POSITION OF PROVINCIAL HOSPITALS.

It is a relief to turn from the somewhat bewildering statistics prepared for the Council of the Metropolitan Sunday Fund, to the more reliable data for comparison obtained by analysis of the reports of the best-known provincial hospitals, accompanied with lucid explanations from those in authority, with respect to what otherwise might appear financial discrepancies. There can be small doubt that these institutions possess numerous advantages over the London hospitals, on account of a more exclusive interest being taken in their welfare by the local population. Each town of any magnitude contains its voluntarily-supported hospital, generally called infirmary, which rich and poor alike are proud of, and feel it incumbent on them, as citizens, to support. In larger cities, such as Liverpool and Glasgow, the growth of population has necessitated the erection of new hospitals in the heart of populous localities, and these, again, have little difficulty in acquiring that solid maintenance which has always been given to the parent establishments. There is even some reason to fear that the craze for special hospitals, which is so frequently condemned as an unnecessary burden on the London hospital system, is finding its way into many of the large towns, especially such as have medical schools attached, but it will be a long time before they reach the protean character of their London models. It can be readily understood that, in consequence of the interest being concentrated in the one great charity of the town and district, greater attention is devoted to minute details of management by executive authorities acting under a body of contributors who, in point of fact, represent the whole local population. Any questionable expenditure is open to comment, and it may be gathered from the annual reports of these institutions that there is a healthy rivalry going on between them, and a disposition in most to take credit for their low average bed cost. With the view of arriving at an approximate estimate of this, we put ourselves in communication with thirteen of the most important provincial hospitals, and are indebted to the official authorities for their reports and explanations with respect to figures which frequently were difficult to reconcile with those quoted from the published records. The analysis extends over three years ending last December, except in the case of the Liverpool Infirmary, now undergoing reconstruction, and whose accounts embrace a period of three years prior to 1887. In striking the average bed cost in each of these institutions, a considerable number, larger than we anticipated, exclude from their calculations an ideal sum for the treatment of out-patients. Some go further than this, and omit expenditure for current repairs and alterations, and replacement of furniture and hospital machinery, which clearly come within the pale of ordinary outgoings. To arrive at anything approaching reliable data, the estimates given are based on the total expenditure of each institution, excluding the cost of new buildings, available balance at the end of the year, and the investment or reinvestment of monies. The same system was applied in the case of the London charities, and when made with the mean number daily resident in the individual hospital, as a divisor, is the only one which places the various establishments on a uniform footing.

| | Total Number of Beds. | 1885. Average Number Daily Resid. | 1886. Average Number Daily Resid. | 1887. Average Number Daily Resid. |
|---|---|---|---|---|
| Edinburgh Royal Infirmary ... | 670 | ... 595 | ... 605 | ... 632 |
| Glasgow Royal Infirmary ... .. | 542 | ... 503 | ... 486 | ... 493 |
| Glasgow Western Infirmary ... | 400 | ... 368 | ... 373 | ... 364 |

NOMINAL AND OCCUPIED BEDS.—(*Continued.*)

| | Total Number of Beds. | 1885. Average Number Daily Resid. | 1886. Average Number Daily Resid. | 1887. Average Number Daily Resid. |
|---|---|---|---|---|
| Manchester Royal Infirmary ... | 300 | ... 253 | ... 253 | ... 259 |
| | | 1884 | 1885. | 1886. |
| Liverpool Royal Infirmary ...... | 257 | ... 218 | ... 214 | ... 220 |
| | | 1885 | 1886. | 1887. |
| Liverpool Royal Southern Hos. | 200 | ... 161 | ... 168 | ... 173 |
| Birmingham General Hospital | 280 | ... 237 | ... 246 | ... 251 |
| Birmingham Queen's Hospital | 120 | ... 112 | ... 113 | ... 113 |
| Leeds General Infirmary......... | 320 | ... 250 | ... 268 | ... 275 |
| Bristol Royal Infirmary ......... | 264 | ... 225 | ... 228 | ... 201 |
| Bristol General Hospital......... | 150 | ... 130 | ... 134 | ... 136 |
| Newcastle-on-Tyne Infirmary... | 270 | ... 234 | ... 257 | ... 241 |
| Brighton Sussex County Hos. | 178 | ... 134 | ... 137 | ... 131 |

AVERAGE COST PER OCCUPIED BED IN THE INSTITUTIONS ABOVE-NAMED, DEDUCTING SHILLINGS AND PENCE WHEN BELOW 10s. :

| | 1885. | 1886. | 1887. |
|---|---|---|---|
| Edinburgh Royal Infirmary ......... | £56 | ... £55 | ... £55 |
| Glasgow Royal Infirmary ......... .. | 49 | ... 49 | ... 47 |
| Glasgow Western Infirmary......... | 51 | ... 48 | ... 49 |
| Manchester Royal Infirmary ...... | 67 | ... 65 | ... 64 |
| | 1884. | 1885. | 1886. |
| Liverpool Royal Infirmary ......... | 55 | ... 57 | ... 55 |
| | 1885. | 1886. | 1887. |
| Liverpool Southern Hospital ...... | 50 | ... 43 | ... 42 |
| Birmingham General Hospital...... | 62 | ... 60 | ... 60 |
| Birmingham Queen's Hospital...... | 72 | ... 69 | ... 67 |
| Leeds General Infirmary ............ | 63 | ... 57 | ... 59 |
| Bristol Royal Infirmary............... | 53 | ... 50 | ... 61 |
| Bristol General Hospital ............ | 58 | ... 55 | ... 58 |
| Newcastle-on-Tyne Infirmary ...... | 59 | ... 53 | ... 49 |
| Brighton Sussex County Hospital | 73 | ... 76 | ... 74 |

With the one exception of the Brighton Hospital, all the above are associated with medical schools of varying extent, and it will be seen that the mean annual cost per occupied bed in all does not exceed £56. The Edinburgh Royal Infirmary, by far the largest educational hospital in the kingdom, with its thousand students engaged in daily occupation in the wards, scarcely reaches the sum named, and the Glasgow hospitals score considerably below it. The scale, embracing the period of three years, ranges from a maximum of £74 at the Brighton Hospital, to a minimum of £45 at the Royal Southern at Liverpool, and some show considerable differences in the cost from year to year, which however, in most instances, is capable of explanation. Of course the most striking feature of the returns is the relatively small cost at which the institutions are conducted when compared with metropolitan hospitals. It is not very easy to explain this, unless on the grounds that the patients are better cared for in London than in the provinces, an inference not likely to be concurred in by provincial hospital authorities. Mr. Moore, the general superintendent of the Royal Infirmary, Liverpool, who has had many years' experience of the working of London hospitals, is inclined to attribute the less cost to the cheaper provisions, fuel, and light met with in the north, but this would scarcely account for the great discrepancy. In the Newcastle-on-Tyne Infirmary there is a marked reduction amounting to £10 a bed in the course of the three years, which is attributed by the house governor mainly to a diminution in the butcher's bill for meat used in the preparation of beef tea and a less lavish employment of medical stores. The accounts for the year 1885 were further handicapped by an expenditure for furniture for a new addition to the building, which had the effect of increasing the bed cost of the year by £2 10*s.*, which ought to have been charged to a special fund. It may be in the recollection of some of our readers that about two years ago a special committee was appointed by the governors of this hospital to inquire into the management and finances of the institution, and a suggestive report of their proceedings appeared in a special supplement of this journal in February, 1887. Although the investigation failed to discover anything amiss with the housekeeping of the establishment, it has apparently done good by diminishing waste in the two directions stated, and it would doubtless be to the advantage of every institution if a similar investigation was made periodically into its internal economy by a competent committee. On the other hand, it is far from our intention to draw an invidious distinction between one hospital and another on account of relative expenditure. It may be that the more costly system gives advantages to the patients which the more economical does not possess, and it would be manifestly unfair on this ground alone, if on no other, to institute a comparison between them. At the same time the advantages of the more bountiful system ought to be known and explicitly stated in the annual reports of such institutions as indulge in it. Apart from this, it is gratifying to notice that in nearly all the establishments quoted there is a decrease in the expenditure during the last three years. This is no doubt attributable in a measure to a reduction in the prices of the main articles of consumption, and in several hospitals the secretaries refer it to a decrease also in the employment of drugs and surgical dressings of an elaborate and costly character. In the lowest average quoted, that of the Liverpool Southern Hospital—there is a distinct fall in cost amounting to £8 a bed since 1885, which the secretary attributes partly to these causes, and also to the fact of the hospital having been furnished with new laundry apparatus in the course of that year. It might be reasonably suggested that renewals or substitutions of the kind referred to should rank with extraordinary expenditure in estimating average cost, but nearly every hospital has to contend every year or two with similar outgoings. Painting, renovating, reflooring, new boilers, renewal of kitchen and laundry apparatus, internal structural alterations, are one or other matters of perennial occurrence, and it is only by including such in our calculations and striking the average cost over a series of years that we can arrive at anything like definite data for comparison. The case of the Bristol Infirmary is almost the only one in the above list which shows a distinct increase in cost, amounting to £10 a bed in its annual expenditure on patients. This is clearly accounted for in the last report of the institution by the closure of part of the hospital for repairs, thereby reducing the average occupied accommodation by 27 beds in the course of the past year. It is a question whether these occasional fluctuations in numbers, peculiar to many hospitals, could not be held in check by always keeping in reserve one ward to which patients might be transferred while others were under repair. It seems an arbitrary proceeding to close a part or the whole hospital for several months, when the work could be done piecemeal without seriously crippling the operations of the establishment. In future articles we propose reverting to the case of the metropolitan hospitals.

